# Do Animals Perceive Human Developmental Disabilities? Guinea Pigs’ Behaviour with Children with Autism Spectrum Disorders and Children with Typical Development. A Pilot Study

**DOI:** 10.3390/ani9080522

**Published:** 2019-08-02

**Authors:** Marine Grandgeorge, Elodie Dubois, Zarrin Alavi, Yannig Bourreau, Martine Hausberger

**Affiliations:** 1 Univ Rennes, Normandie Univ, CNRS, EthoS (Éthologie Animale et Humaine)—UMR 6552, F-35380 Paimpont, France; 2 Centre de Ressources Autisme, CHRU of Brest, Hospital of Bohars, F-29200 Bohars, France; 3 Inserm, CIC 1412, CHRU of Brest, F-29200 Brest, France; 4 Univ Rennes, Normandie Univ, CNRS, EthoS (Éthologie Animale et Humaine)—UMR 6552, F-35000 Rennes, France

**Keywords:** interaction, interspecific, guinea pig, behaviour, autism spectrum disorders, children, animal assisted intervention

## Abstract

**Simple Summary:**

Many professionals argue that animals in animal-assisted interventions are able to perceive people’s developmental disabilities and to adapt to them. To date, there is no scientific evidence in support of this hypothesis. Humans and animals use cues to modulate their interspecific interactions. However, the potential underlying mechanisms for these cues are unknown. Thus, we hypothesized that animals could perceive particular behaviours such as developmental disabilities present in children. To test this hypothesis, guinea pigs’ or GP behaviours were compared between two groups of children (*N* = 44, aged between 6 to 12 yo) with typical development (TD) and with autism spectrum disorders (ASD). We evidenced that GP behaviours in the presence of children differed slightly when encountering ASD children versus TD children: more positive behaviours toward ASD children at the onset, and more feeding and resting in the presence of TD children toward the end of an interaction. One could explain this by GP curiosity toward ASD children behaviours (e.g., no marked behaviours such as attempts to touch), whereas GPs seemed calmer at the end with TD children (i.e., interacting with ASD children may be a little stressful). This partly gave support to our study’s hypothesis. GPs seemed to perceive developmental disabilities during a first encounter with children and to adjust their behaviours to that of children. We discuss the issues of animal training, animals’ well-being and acute stress, whether they are pets or used in animal-assisted interventions, and we propose perspectives that would help further our understanding.

**Abstract:**

Some cues used by humans and animals during human-animal interactions may have significant effects, modulating these interactions (e.g., gaze direction, heart rate). This study aimed to determine whether an animal in human-animal interactions is capable of “perceiving” its human partner’s potential developmental “disabilities”. To test this hypothesis, we studied guinea pigs (GP) behaviours in the presence of 44 6-to-12-year-old children with either typical development (TD children) or with autism spectrum disorders (ASD children). Thus, we recorded the GP behaviours during the entire session (to establish their time budget) and focused in particular on the onset and end of physical interactions. The GP behaviours (e.g., feeding, resting, self-grooming, exploring) were not significantly different between the two groups of children during the whole session. GP behaviours in the presence of children differed slightly when encountering ASD children versus TD children: more positive behaviours toward ASD children at the onset, more feeding and resting in the presence of TD children toward the end of an interaction. TD children showed longer-lasting interactions. One could explain this by GP curiosity toward ASD children behaviours (e.g., no marked behaviours such as attempts to touch), whereas GPs seemed calmer at the end with TD children (i.e., interacting with ASD children may be a little stressful). This partly gave support to our study’s hypothesis. GPs seemed to perceive developmental disabilities during a first encounter with children and to adjust their behaviours to that of children. We discuss the issues of animal training, animals’ well-being and acute stress, whether they are pets or used in animal-assisted interventions. Further studies (on pets or animal-assisted interventions) are warranted.

## 1. Introduction

Each individual has its own way to collect, perceive, decode, interpret and emit cues during interactions with its conspecifics. An animal’s interactions with its conspecifics vary according to its species and past experience [1]. As conspecifics represent an essential part of the environment of group-living individuals, humans can be considered a major element of domestic animals’ immediate environment [2]. During the first encounter between a human and a pet (e.g., a cat), physical contact was reported as the key event [3]. It has been confirmed in several studies that most interactions between humans and other mammal species are tactile contacts (with dogs [4], cats [3], rabbits [5], as well as guinea pigs (GP) [6,7]). Cues given by tactile interactions as well as other cues used by humans and animals in such contexts may have a significant effect, enabling them to modulate their interactions. Some companion animals, in particular dogs, are able to perceive and decode human gaze direction, general postures, gestures or odours in different situations [8]. Similarly, humans can perceive and decode some of the cues displayed or emitted by animals in order to adapt their interactions with them (e.g., body postures, vocalisations [9,10]). Such cue decoding is associated with an animal’s level of bonding or acquaintance with its human partner. For example, Sankey et al. [11] showed that horses respond to the familiar order similarly to attentive familiar and unfamiliar humans, but they monitored much more the unfamiliar person’s behaviour by turning their head and gazing at him. Similarly, GP behaviour in animal assisted interventions (AAI) is influenced by the professionals as well as by the patients characteristics [6]. Moreover, visual attention influences human-animal short- and long-term interactions (e.g., this was shown in human-horse interactions [12]). The way humans behave can have a direct impact on an animal’s perception of the situation, and hence its representation of humans (e.g., this was shown when training horses [13]). Many AAI professionals suggest that animals are able to perceive people’s developmental disabilities and to adapt to them [14]; however, to date, no scientific data exist. Moreover, interspecific interactions are not always positive. For example, a misconception could lead to ending an interaction (e.g., in human-dog interactions [15,16]) or to serious events such as dog biting, cat scratches or injuries caused by a horse (e.g., [17,18,19]). Interspecific interaction failures are most often the result of a misconception of the other species’ behavioural cues as well as of that species’ needs. For example, Keeling et al. [20] showed that being nervous (measured by the heart rate) influences horse–human interactions. Moreover, many authors have hypothesized a significant impact of humans’ unintentional behavioural approaches on animals’ behavioural responses, yet only few tested it. For example, Sankey et al. [16] reported disturbances in the horses’ response to an order when the human was inattentive (e.g., the eyes were not visible or not directed at the horse). Currently, animals are part of humans’ lives and seem to have several different effects. As companion and co-therapist animals, they contribute to enhance humans’ physical, emotional and/or psychological well-being [21,22,23]. People ranging from young to elderly—with or without disabilities—in both experimental and home settings were observed to benefit from contact with different animal species (e.g., with dogs, cats, horses, rodents [24]). Such reported beneficial effects on humans have not been observed on animals when encountering humans. Recent but rare reports showed that animal-assisted interventions may stress the animals (e.g., horses, dogs, GPs [6,14,25]) in relation to the humans’ individual characteristics (e.g., age for dogs [26]). Moreover, the modalities and nature of interactions between humans and animals remain poorly understood in such contexts [27,28,29]. For example, in AAI, GP behaviour is influenced by the mere presence of humans (compared to the control situation without humans: they freeze more frequently, spend less time eating and vocalising), as well as by the presence of retreat possibilities [6]. Autism spectrum disorders (ASD) are some of the most frequently reported disorders for which interacting with animals as co-therapists or companions [30] can benefit ASD individuals (e.g., interactions with dogs, horses, GP [31,32]). ASD individuals are characterized by their difficulty in establishing social interactions and communicating, for instance to establish and/or maintain exchanges, as well as their inability to initiate an interaction or to adjust to different social expectations [33]. They also present nonverbal communication problems such as abnormal eye contacts, postures or gestures, and they are unable to understand them. People with ASD (ASD-people) display restricted and repetitive behaviours and present particular sensory characteristics, i.e., hyper- or hypo-reactivity to sensory inputs or an unusual interest in sensory aspects of the environment [34,35]. ASD-people’s visual recognition of biological motion is impaired [36], and their motion integration is deficient, leading to various levels of difficulties to perceive and integrate environmental dynamic multisensory stimuli [37]. Authors argue that these developmental disabilities have implications for socio-emotional interactions or motor skills, thus affecting everyday skills [38,39,40]. Taken all together, these elements question the possible impact of these ASD behavioural characteristics on communication with animals. Redefer & Goodman [41] proposed that the simple repetitive non-verbal behaviours of companion animals were easier to decode for ASD-people than humans’ behaviour. This could be explained partly by specific animal face processing focused on eyes (this is not the case for human faces [42]). Previous studies have shown that the behaviour of ASD children with humans differs in the presence of animals such as GPs [29,43] or dogs [28,44]. For instance, in the presence of a human, a dog and objects, they show a greater interest in the dog than in the human or the objects [28]. Comparing the behaviour of ASD children to that of children with typical development (TD children), authors reported that a third of the ASD children were not interested in the GPs and that ASD children were at a greater mean distance from the GPs than the TD children were [45,46]. In conspecific and interspecific contexts, an interaction may result from the following. ‘A’ component has an action X on ’B’ component or ‘A’ component does X to ‘B’ component and ‘B’ component responds with action ‘Y’ [47]. We believe that all individual components (e.g., humans or animals) involved in the interaction should be assessed. To date, most studies have focused on the human component of the animal-human interactions. To bridge the knowledge gap on the animal component of the animal-human interactions, we designed this study.

The aim of the present study was to evaluate, during the first encounter, the behaviour of a companion animal, here a GP, according to the behavioural characteristics of its partners, i.e., ASD children and TD children. These data will help us evaluate the common preconception that animals can perceive human developmental disabilities.

## 2. Materials and Methods

### 2.1. Study Design

Our design based on video recordings and data were collected in the children’s homes between Summer 2008 and Spring 2009.

#### 2.1.1. Participants

Four brown long-coat adult female guinea pigs (*Cavia porcellus)* (GPs) were used. They had been purchased at an early age (around 8-weeks old) from a pet shop and raised in a family setting until they were involved in this study. Each animal was handled positively on a daily basis (at least 30 min per day, every day) and lived alone in its cage. This training was maintained during the study. Only healthy and non-aggressive animals were selected. To avoid potential stress or weariness, each GP was used for a maximum of three sessions per day. We analysed the data only for GPs for which we had a full 15-min video-recording (i.e., some of the recordings were stopped by the child playing with the camera) representative of the whole session. Thus, 44 sessions involving three GPs were included in this study from the initial pool of 90 sessions [45].

Forty-four children were included in this study. The first group included 22 TD children (8 ♀/14 ♂, mean age ± SD: 9.4 ± 0.4 years old). They were recruited by announcement and on a voluntary basis. They attended school regularly; none met the diagnostic criteria for ASD or other pervasive developmental disorders according to the parental report. The second group included 22 ASD children (22 ♂, mean age ± SD: 9.3 ± 0.4 years old). All received their diagnosis of ASD from the *Centre de Ressources Autisme Bretagne*, a specialist diagnosis institution. Their diagnosis was based on the DSM IV [48] and the ICD-10 [49] criteria and was confirmed using the ADI-R (Autism Diagnostic Interview-Revised [50] (mean total score of 40.3 ± 4.7, min-max: 16–56). Vineland Adaptive Behaviour Scales [51] (*n* = 16, communication X = 30.7 ± 9.8 months, daily living skills X = 35.0 ± 6.7 months, and socialisation X = 28.3 ± 7.1 months) and the Childhood Autism Rating Scale questionnaire [52] (*n* = 16, total score X = 34.1 ± 2.5) were used to conclude the assessment, when necessary. These children were diagnosed mainly with mild ASD according to these tools.

#### 2.1.2. Ethical Note

The data presented in this study originated from a study performed in 2008–2009 (described in [45]). The study was conducted in accordance with the French regulations governing the care and use of research animals. The animals used in this study were considered as companion animals used in observational studies; no specific law existed in France at that time. To ensure the well-being of the animals, they lived in a family context and were positively handled every day; each GP was used for a maximum of three sessions per day, and 6 per week, and they were frequently and regularly examined by veterinarians during the study. All were definitively adopted by their host family after the end of the study.

All children were accompanied by one of their parents during the test. The present research was observational, non-invasive and did not involve any pharmacological interventions. According to the current French laws on the protection of persons in biomedical research (law No 88-1138, the so-called Huriet-Serusclat law of the 20 December 1988, amended in 2004—law of the 9 August 2004), such protocols do not require the approval of an ethics committee. Hence, in accordance with these laws, parents only had to give an informed written consent to allow their child’s participation in the study and to film their child prior to their inclusion in the study. They mentioned the use of video they authorized (i.e., none except for analysis for the research; only for research purposes; both for research and teaching purposes). All children also verbally approved their participation. After all sessions had been performed, the videos were anonymised, being attributed a number, and they were stored in a locked box. At any time, even after the end of the research, they and their child had the right to withdraw. The risks were considered as minimal and mainly concerned potential violent gestures toward GPs, being bitten by the GP or an expression of fear in the child. If one of these events happened, the session was stopped immediately.

### 2.2. Study Design

#### 2.2.1. Equipment

The pet device included a standard cage (70 × 40 × 20 cm) cleaned before each session. To facilitate interactions, the pet’s shelter and the cage top were removed. The cage floor was covered with sawdust—cleaned before each session. Water and food (commercial pellets and hay) were provided *ad libitum*. The open cage was placed on a low table.

Both the animals’ and the children’s behaviours were recorded using two video cameras, one mounted on a tripod and facing the cage (focusing on the animal’s behaviour) and the second one carried by the observer (focusing on the child’s behaviour).

#### 2.2.2. Study Context

An appointment for one hour was set between the observer and the family at least 2 weeks before the session. Both populations of ASD and TD children were studied simultaneously. The children were randomly assigned to sessions. All sessions were performed at the children’s home. Thus, the GP was transported in its cage by car (duration of journey: from 5 to 60 min). To minimize the effects of transportation on the GP’s behaviour, we left the animal alone for at least 20 min between arriving at the child’s home and beginning the session.

During the session, two adults were present in the room: one parent and the observer. The parent present during the session was usually the child’s mother, except for single father families or when the mother was temporarily absent. All tests were performed by the same female observer (MG).

#### 2.2.3. Procedure

Before setting up the session, the observer instructed the child and his/her parent and, addressing both the child and parent, we stressed that no behaviour was considered either right or wrong.

- We told the child that during the session he/she could behave as he/she wanted. For example, he/she was free to interact (or not) with the unfamiliar animal;
- We asked the parent to sit at some distance from the cage, to stay neutral and silent (e.g., no encouragement, no smiling at the child) during the session. The parents of ASD children were asked to confirm that their child had heard and understood the instructions.

After making sure that the instructions had been understood, the equipment described above was installed. When all of the equipment was installed, the observer then asked the child and the parent to come into the room. As soon as they entered, the observer switched on both cameras.

The observer remained neutral and silent in an unobtrusive place in the room; she moved only if absolutely necessary to avoid losing view of the child’s front part of the body (e.g., when a child had his/her back to the observer) and stopped the session after 15 min.

Only the data recorded by the video camera focused on the animal are presented in this paper, and the GPs’ behaviours were analysed as described below.

### 2.3. Data Collection and Analyses

The data analyses were based on the 15-min video recordings. Here, we focused on the behaviours in the cage area. The observation of behaviour and the recording of events were performed by two raters blind to the child’s diagnosis. Both raters had previous experience in coding human-animal interactions. The coding procedure was divided into two steps. First, there was a training part when two raters coded half of the videos together. Then, the other half of the data was coded by only one rater.

#### 2.3.1. GP Behaviours

*Time budget of GP.* The first step of our study was to establish the time budget of GPs during a session in the presence of the child (ASD or TD). A time budget indicates the proportion of time that animals or humans spent in different behaviors, or in performing different behavioural categories. Therefore, the GP’s behaviour was analysed by instantaneous scan sampling using 10-s intervals, yielding 90 scans per session [53]. Our behavioural repertoire was based on previous research on GPs [6,54,55,56,57] and was adapted to our aim. We recorded maintenance behaviours, behaviours directed toward the child, and other behaviours, such as exploration and locomotion (Table 1). Thus, we obtained frequencies (in % of scans) of the different behavioural items that were recorded.

Onset and end of physical interactions. During the second step, we focused on two time periods: the onset and the end of each physical interaction. Given that most interactions (> 50%) were not entirely visible (e.g., when the GP was hidden by the child), we only focused on these two time periods. First, the onset of an interaction was defined as the first behaviour of a GP and a child following physical contact between them. Second, the end of an interaction was defined as the moment when a GP behaviour or a child’s behaviour induced an interruption of their physical contact (≥ 1 s), that is the last behaviour before the end of the interaction. An interaction refers to a sequence where A performs behaviour X toward B and B responds with behaviour Y, thus involving two individuals displaying one or more types of behaviours [1,47]. In the present case, the first and last behaviours performed by a GP during an interaction were noted. Only tactile interactions were recorded here (Table 1), regardless of any association with gazes. In addition, at each onset and end of an interaction, we noted whether the GP was the initiator of the onset/end and the vocalisations by the GP. The procedure seemed to privilege the initiation by the child (the GP was constrained by the cage), but the GP could initiate if the child let his/her hand into the cage without touching the GP. The behaviours were grouped in three general categories: GP positive behaviours toward a child (i.e., approaching and exploring a child), GP negative behaviours toward a child (i.e., avoiding, retreating and jumping) and GP maintenance behaviours (i.e., feeding, self-grooming, body shaking and resting/sleeping) [56].

The data concerning the GP time budgets and the frequencies of the GP behaviours during the onset and the end of physical interactions were compared between the two groups (ASD and TD).

#### 2.3.2. Children’s Behaviours

The children’s behaviour data were analysed only when the GP behaviours were significantly different between the two groups of children:

- When the GP time budgets differed, the children’s behaviours were analysed using instantaneous scan sampling every 10 s [53].
- When the behaviours at the onset and end of physical interactions with the GP differed, the children’s behaviours were analysed using continuous sampling (Table 2).

### 2.4. Statistical Analyses

The data were analysed using Minitab 15© and IBM SPSS Statistics 19©. As our data did not fit a normal distribution, we applied non-parametric statistical tests [58]. The significance threshold was *p* = 0.05. Mann–Whitney U tests were used in order to compare two independent samples (e.g., the difference in behaviours between the two groups of children). Kruskall–Wallis tests and Mann-Whitney U-tests were used to compare the differences in behaviours among the three GPs used.

## 3. Results

The data analysis included 4 h, 39 min and 7 s of video recordings, excluding non-visible elements (e.g., dead angle, GP hidden by the child). Of the 44 children, only 4 ASD-children never approached the GP. Thus, all analyses were based on 18 ASD children and 22 TD children. We recorded the same frequencies of GP interaction sequences during the 15-min session for ASD children and for TD children (n_ASD_ = 18, n_TD_ = 22, $\bar{X}$_ASD_ = 11.9 ± 3.7, $\bar{X}$_TD_ = 12.5 ± 4.4, U = 399, *p* = 0.899). On average, the TD children-GP interactions lasted significantly longer than the ASD children-GP interactions (n_ASD_ = 18, n_TD_ = 22, $\bar{X}$_TD_ = 32.1 ± 0.4 s, $\bar{X}$_ASD_ = 22.6 ± 0.6 s, U = 34.4, *p* = 0.017). No significant differences in frequencies or durations were found between the GPs (Kruskall–Wallis tests *p* > 0.05).

### 3.1. Time Budget of GP

Eight types of GP behaviours were recorded (Table 3). For example, no freezing was observed. The GP behaviours most frequently observed were resting ($\bar{X}$ = 49.6 ± 12.0%), feeding ($\bar{X}$ = 26.6 ± 11.4%) and exploration ($\bar{X}$ = 16.7 ± 7.2%). No difference was observed in the GPs’ behaviours according to the children’s group (all Mann-Whitney-U tests *p* > 0.05) or the GP (all Kruskall-Wallis tests *p* > 0.05).

### 3.2. Onset of an Interaction

As expected, given the procedure, most interactions were initiated by the children ($\bar{X}$_GP_ = 3.6 ± 8.0%, $\bar{X}$_children_ = 96.4 ± 8.0%; Z = 5.19, *p* < 0.001). ASD children initiated as many interactions as TD children (all tests *p* > 0.05). There was a higher variability in the number of interaction initiations by GPs when encountering ASD children than TD children ($\bar{X}$6.7 ± 11.5% vs. $\bar{X}$0.7 ± 0.9%; U = 404.5; *p* = 0.552).

#### 3.2.1. Guinea Pigs

**Figure 1:**
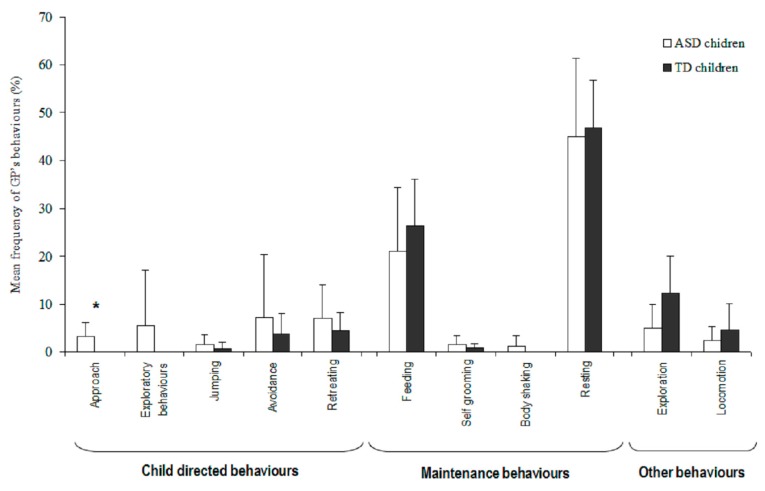
GP’s behaviours at the onset of an interaction (in percent) with ASD children (white) and TD children (black). Level of significance: * *p* < 0.05 (Mann Whitney-U test).

At the onset of an interaction, most GPs were resting ($\bar{X}$ = 46.0 ± 13.2%) or feeding ($\bar{X}$ = 23.9 ± 11.4%), and other behaviours were rarely observed (Figure 1). When these behaviours were compared according to the children’s groups, the GPs approached ASD children more frequently than TD children (U = 413, *p* = 0.023). No statistical differences were observed for the other GP behaviours according to the children’s groups (all Mann-Whitney-U tests *p* > 0.05).

GPs displayed more positive behaviours (i.e., approaching and exploring a child) toward ASD children than toward TD children ($\bar{X}$_ASD_ = 7.1 ± 10.2%, $\bar{X}$_TD_ = 0.0 ± 0.0%; U = 42.4, *p* = 0.0099). No such differences were found for the other behavioural categories (negative: $\bar{X}$_ASD_ = 12.7 ± 11.8%, $\bar{X}$_TD_ = 9.0 ± 6.6%; U = 383, *p* = 0.686; maintenance: $\bar{X}$_ASD_ = 42.9 ± 16.8%, $\bar{X}$_TD_ = 60.0 ± 8.7%, U = 305, *p* = 0.08). No significant statistical difference was found in the GP behaviours between the two groups of children (all Kruskall-Wallis tests *p* > 0.05).

GP vocalisations were recorded during 1.1 ± 0.9% of the interactions’ onset, with no difference according to the children’s group ($\bar{X}$_ASD_ = 0.6 ± 0.9%, $\bar{X}$_TD_ = 1.50 ± 0.9%, U = 311.5, *p* = 0.08) or GPs (both Kruskall-Wallis tests *p* > 0.05).

#### 3.2.2. Children

As some of the GP behaviours differed according to the group of children, the children’s behaviours were analysed in order to better understand the GP behaviours.

**Figure 2:**
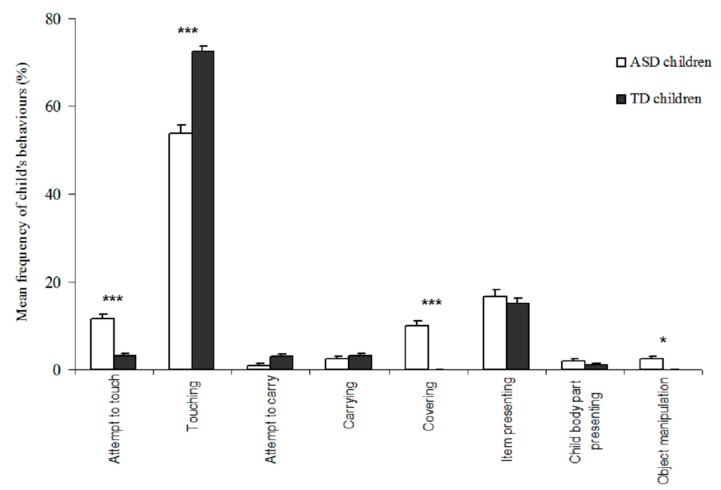
Behaviours of the ASD- and TD children during the onset of an interaction. Level of significance: *** *p* < 0.001, * *p* < 0.05 (Mann-Whitney U-tests).

The children displayed different types of behaviours (Figure 2). Most interactions began with touching the GP ($\bar{X}$ = 63.1 ± 1.5%), presenting an item ($\bar{X}$ = 15.8 ± 1.5%) and attempting to touch ($\bar{X}$ = 7.3 ± 0.8%). Interestingly, all attempts to touch were unsuccessful because children withdrew their hand just before touching. TD children touched the GP more frequently than did ASD children (U = 39.4, *p* < 0.001), whereas ASD children attempted to touch the GP more frequently than did TD children (U = 41.5, *p* < 0.001). Some ASD children initiated contact by covering the GP with sawdust, whereas none of the TD children did this (U = 32.7, *p* < 0.001). Some ASD children manipulated objects, whereas none of the TD children did (U = 31.2, *p* = 0.012).

### 3.3. End of an Interaction

Most interactions were ended by the child ($\bar{X}$_children_ = 68.5 ± 6.4%, $\bar{X}$_GP_ = 31.5 ± 6.4%; U = 47.8, *p* = 0.021). No significant differences were found between the ASD- and TD children groups according to the GP behaviour (both Kruskall-Wallis tests *p* > 0.05). No significant differences were found between the two groups of children regardless of the role of the child ($\bar{X}$_ASD_ = 60.7 ± 15.7%, $\bar{X}$_TD_ = 74.9 ± 8.8%; U = 320.5, *p* = 0.191) or GP ($\bar{X}$_ASD_ = 39.3 ± 15.7%, $\bar{X}$_TD_ = 25.1 ± 8.8%; U = 471.5, *p* = 0.191) in ending the interaction.

#### 3.3.1. Guinea Pigs

**Figure 3:**
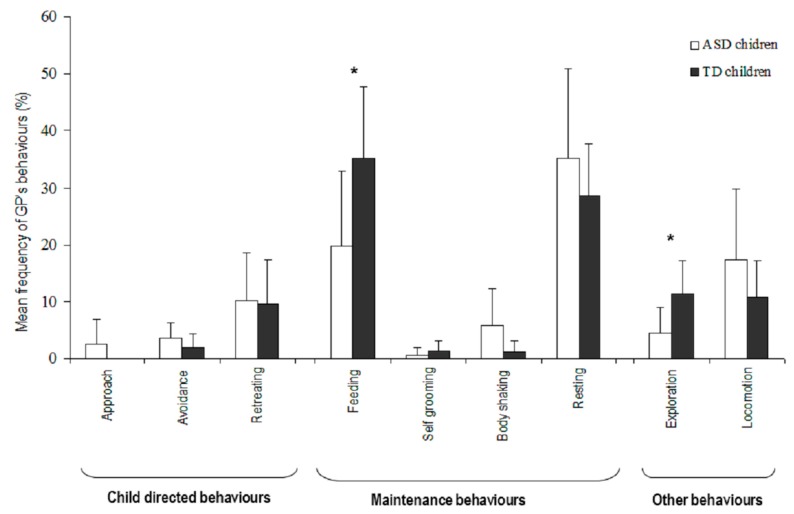
GPs’ behaviours at the end of an interaction, in the presence of ASD children (white) and TD children (black). Level of significance: * *p* < 0.05 (Mann Whitney-U test).

GPs spent most of their time resting ($\bar{X}$ = 31.6 ± 12.4%), without any significant difference according to the group of children (U = 375.5, *p* = 0.87), but the time spent feeding ($\bar{X}$ = 28.2 ± 13.2%) differed significantly according to the children’s group ($\bar{X}$_ASD_ = 35.2 ± 15.5%, $\bar{X}$_TD_ = 28.6 ± 9.1%; U = 297.5, *p* = 0.049) with more time spent feeding in the presence of TD children (Figure 3).

Moreover, GPs explored more in the presence of TD children than in the presence of ASD children ($\bar{X}$_TD_ = 11.4 ± 5.7%, $\bar{X}$_ASD_ = 4.5 ± 9.0%; U = 300, *p* = 0.044). Other behaviours (e.g., locomotion) were observed less frequently, and their frequencies did not differ significantly between the two groups of children (all Mann-Whitney-U tests *p* > 0.05). These frequencies were not influenced significantly by the presented GP, regardless of the group of children (all Kruskall-Wallis tests *p* > 0.05).

Neither the children group appurtenance nor the individual GP significantly influenced the frequencies of the three behavioural categories (negative, U = 390.5, *p* = 0.534; positive, U = 391, *p* = 0.121; maintenance, U = 340, *p* = 0.437; all Kruskall-Wallis tests *p* > 0.05).

GPs vocalised during the final phase of 1.3 ± 1.1% of the interactions, and neither group of children ($\bar{X}$_TD_ = 1.7 ± 1.2%, $\bar{X}$_ASD_ = 0.7 ± 0.8%, U = 319, *p* = 0.121) significantly influenced the vocalisation frequencies, and neither did the individual GPs (both Kruskall-Wallis tests *p* > 0.05).

#### 3.3.2. Children

Most children ended an interaction by withdrawal (e.g., their hands) ($\bar{X}$ = 89.1 ± 1.3%), especially TD children ($\bar{X}$_TD_ = 92.4 ± 5.8%, $\bar{X}$_ASD_ = 85.0 ± 7.8%, U = 50.9, *p* = 0.005). ASD children tried to start an interaction more often than TD children did ($\bar{X}$_TD_ = 4.1 ± 4.3%, $\bar{X}$_ASD_ = 12.6 ± 7.2%, U = 51.4, *p* < 0.001). No difference in putting down or repelling the GP was observed between the groups of children (both Mann-Whitney U-tests *p* > 0.05). Interestingly, no child was resting at the end of an interaction.

## 4. Discussion

Our aim was to explore if animals could “perceive” developmental disabilities during a first interspecific encounter and adjust their behaviours accordingly. The little but significant differences we found partly gave support to our study’s hypothesis. Even if ASD children, as well as TD children, remained the principal initiators of the interactions and the GP did not initiate or end more interactions with ASD children than with TD children, we found that GP behaviours in the presence of children differed slightly when encountering ASD children versus TD children: there were more positive behaviours toward ASD children at the onset, and more feeding and resting in the presence of TD children toward the end of an interaction. One could explain this by (1) GPs’ curiosity toward ASD children behaviours (e.g., no marked behaviours such as attempts to touch), which differed from TD children behaviours; and (2) GPs seemed more calm at the end with TD children (i.e., interacting with ASD children may be a little stressful). Taken altogether, GPs seemed to perceive developmental disabilities during a first encounter with children and to adjust their behaviours to that of children.

Our study GP time budget was not consistent with that reported in the literature, except for the time spent resting (here, 49.6 ± 12.0% versus 49%, but only for females [57]). Compared to Fuchs’ study [59] on cavies, our GPs spent less time feeding (26.6% versus 39%) and self-grooming (0.9% versus 4.3%) but more time moving and exploring (1.5% and 16.7% versus 12%). Compared to Gut and colleagues’ study [6] in a comparable situation (therapy with no possibility to retreat), our GPs were for example stroked less (more than 70% [6]) and moved less (i.e., here 1.5% locomotion versus around 10%). These discrepancies may be explained—in part—by the differences in the context of the observational settings, i.e., the presence of a single GP versus a group (although only one was observed), TD and ASD children versus patients and no humans, the setting being at the human’s home versus in a hospital (respectively our study and other authors). Domestic GPs explore less than cavies do, which could be due to their man-made housing environments [59]. However, Rood [55] explained that when a GP felt insecure, e.g., when exploring an unfamiliar environment, it was able to enhance its locomotive behaviour. Their locomotion is also reduced by the presence of humans [6]. Thus, our GP time budget may indicate that the situation was globally not stressful regardless of the human partner, i.e., an ASD or TD child.

At the onset of an interaction, GPs displayed more positive behaviours, e.g., more approaches, toward ASD children. At the same time, ASD children displayed no marked behaviours or more difficulties in touching (observed as attempts to touch a GP) than TD children did. The relative proximity of the GPs provided more opportunities for GPs to approach the ASD children. In fact, the TD children displayed more touching behaviours. At the end of an interaction, GPs displayed more feeding and exploration in the presence of TD children than of ASD children. One could argue that GPs seemed calmer at the end with the TD children. As an interaction’s ending may reflect the quality of the interaction, we could hypothesis that some of characteristics of ASD behaviours (e.g., at the onset of an interaction: covering a GP with sawdust) might induce a source of discomfort for the animal (observed by a reduced frequency of feeding, for example). What is most surprising was that GPs did not end the interaction more often with ASD children than with TD children. Moreover, no difference was observed concerning GP negative behaviours (i.e., avoiding, retreating and jumping) that could also explain stress, fear or misconception of a child’s behaviour by the animal. Indeed, according to the literature in experimental situations, when a human emits contradictory cues (e.g., pointing at an empty box when this gesture usually indicates where the food is) animals, like dogs, hesitate or sometimes refuse to perform the task [16] and thus end the interaction. In a “natural” environment (i.e., at home), after an agonistic behaviour displayed by 2-to-5 yo children, the dog retreated or went away in most cases [15].

Furthermore, we cannot rule out that these ASD-specific behaviours may also reflect an apprehension of the unknown GP. Indeed, ASD children have higher levels of fear than TD children when encountering specific non-frightening yet unknown animals (e.g., when facing birds and squirrels [60]). This fear could also be expressed in more frequent attempts to touch the animal. These attempts were prematurely ended either before or after touching the GP. ASD children may be more afraid to be bitten by the GP. Indeed, in a large sample, it has been shown that almost 9% of 6-to-12-yo ASD children were afraid of animals in general [61]. Most children are afraid of animals because of their fear of bites [62]. Furthermore, ASD children were reported to display some motor disabilities and an atypical management of space [63,64]. This may explain in part their difficulty in maintaining tactile interactions with GPs. Another non-exclusive explanation may be that the GP’s approach is too quick for ASD children, who have a difficulty interacting with so called “too fast” environments [37]. Reports have previously shown that humans’ anxiety can influence animals’ behaviours (e.g., this was shown with horses [20]). Moreover, a simple interaction with a patient can modify a GP behaviour, especially if it has no possibility to retreat or interact with other GPs [6]. Nevertheless, that study did not mention the patients’ disabilities, which may have been totally different, involving various types of behaviours. Thus, an assumption may be made given that animals adapt their behaviour to that of their partners [11,13].

Finally, our data confirms previous studies showing that tactile interactions are important in human-animal communication [3,65] (i.e., here, around one per minute per child in both groups). Nevertheless, TD children’s tactile interactions lasted longer than those of ASD children, who preferred to stay at a greater distance from the animal [46]. Our results are comparable with those of O’Haire et al. [29], showing that ASD children hand-touched the toys more often than the GPs. ASD children’s tactile interactions may depend on experimental contexts and population. Indeed, Kršková et al. [43] reported frequent ASD children-guinea-pigs tactile behaviours, without any comparison to TD children.

The main limitation of our study was our methodological choice to focus only on the onset and end of the interactions. At the same time, this was the strength of our study for the following reasons. The data collected at the onset and the end of animal-human interactions can have an impact on the course and quality of the interaction, respectively. Moreover, this choice allowed a more comprehensive collection of behaviours. During these two time periods, both the child and GP were clearly visible, while at other times for instance the GP was hidden by the child’s body. This methodological issue should be solved for future studies. As often observed in similar studies, the sample size is one of the other main limitations. We had four GPs but retained only three for methodological reasons. As suggested by Gut and colleagues in a similar study on four GPs [6], the results have to be considered with caution given inter-individual variations in the animals’ behaviour. Nevertheless, our 44 sessions of encounters yielded good preliminary results, especially because the videos were blind analysed (i.e., the rater was not aware of the participant’s diagnosis). We agree with Gut and colleagues concerning the “observed behaviour […] interpreted […] on the basis of existing literature on stress in GP. It is unclear how these interpretations are correlated with the GP perception and physiological or even health or longevity outcomes”. Moreover, animal potential stress due to transport and relocation may have played some part in the overall behaviour, even if it has not been evaluated. The coding procedure of the videos (half of the codings were conducted by two raters, the other half were conducted by another rater) was a further limitation of our study, although the single rating involved both groups of children equally.

## 5. Conclusions

Taken together, GPs seemed to perceive developmental disabilities during a first encounter with children and to adjust their behaviours to that of children. Even if the differences remained understated, one could argue that such abilities could be enhanced with time, when relationships would be established [47] or when animals have acquired an experience of disabled people [6]. Thus, a replication of our results is required with GPs as well as with other animal species. Moreover, we suggest further studies to assess the hypothesis that the animals, e.g., GPs, but also other species with different cognitive and perceptual skills, seek more contact with disabled people, e.g., ASD people, than with TD people. Further studies could also employ a “control” description of GP behaviour in colonies in which the animals are not handled. Given our findings, and taking into account the potential predictors of animal behavior, e.g., animal training, animal well-being, and the presence of acute stress, further studies (on pets or animal-assisted interventions) are warranted.

## Figures and Tables

**Table 1:** Behavioural categories of guinea pigs and their definitions.

Guinea Pig	
Behavioural Category	Definitions
**Maintenance behaviours**	
Feeding	Standing on four feet or lying down eating, for example, hay on the floor or pellets. Here, drinking is included in this behavioural category
Self-grooming	Cleaning the skin or fur of its own body
Body shaking	Moving body to-and-fro with jerky movements
Resting/sleeping	Lying on the floor or resting with eyes closed or almost closed
**Behaviours directed toward the child**	
Approach	Moving toward the child without physical contact
Exploratory behaviour	Moving its head oriented towards the child and sniffing a child’s body part, with or without locomotion
Jumping	Moving or jerking suddenly as a consequence of the child’s behaviour
Avoidance	Keeping away from the child by moving a body part without walking or running
Retreating	Moving away from the child to actively break an interaction by walking, running or thrashing about
**Other behaviours**	
Exploration	Orienting its head toward an item (e.g., pellets) or sniffing or biting with small and repeated bites or using its head to push an object, with or without locomotion
Locomotion	Walking or running in the cage without any exploratory behaviour. All locomotion behaviours were recorded, regardless of their speed and duration.
Freezing	Stopping all movements for a timespan > 1 s.

**Table 2:** Children’s behavioural categories and their definitions.

Child	
Behavioural Category	Definitions
Behaviours Directed Toward the Guinea Pig	
**Actions**	
Attempt to touch	Directing a movement toward the GP * to establish physical contact but stopping before touching
Touching	Establishing physical contact
Attempt to carry	Directing a movement towards the GP to hold, support or take it from one place to another but stopping it before carrying
Carrying	Holding, supporting or taking the GP from one place to another (using one or both hands)
Grasping	Taking hold of or seizing firmly a part of the GP’s body
Covering	Placing something upon or over the GP (e.g., sawdust)
Food presenting	Placing a food item in front of the GP’s head
Item presenting	Placing an item in front of the GP’s head (e.g., a toy)
Child presents part of his/her body	Placing a part of his/her body (e.g., hand) in front of the GP’s head
**Withdrawals**	
Repulsing	Rebuffing or rejecting with rudeness or coldness
Removing	Withdrawing a part of the body (e.g., hand) from the GP
Going back	Moving his/her body backwards from the GP
Putting down	Replacing (or releasing) the GP in its cage area
**Other behaviours**	
Resting	Absence of motion
Object manipulation	Taking an object and manipulating it (e.g., toy).

* GP: guinea pigs.

**Table 3:** Guinea pigs’s behaviours (mean frequency ± SD).

	Feeding	Self-Grooming	Resting	Jumping	Avoiding	Retreating	Exploration	Locomotion
Mean frequency ± SD (%)	26.6 ± 11.4	0.9 ± 0.8	49.6 ± 12.0	3.7 ± 3.6	2.9 ± 2.8	0.6 ± 0.8	16.7 ± 7.2	1.5 ± 1.1
According to guinea pig								
GP1	31.8 ± 9.7	0.9 ± 0.7	45.6 ± 8.5	5.1 ± 5.3	4.0 ± 3.7	0.7 ± 1.3	14.4 ± 4.5	1.2 ± 0.9
GP2	19.1 ± 13.5	1.5 ± 1.0	60.4 ± 15.7	2.7 ± 0.2	1.5 ± 1.0	1.2 ± 0.9	13.6 ± 0.8	2.7 ± 0.2
GP3	10.8 ± 8.8	0.4 ± 0.3	77.5 ± 14.5	1.6 ± 0.9	1.1 ± 1.1	0.5 ± 0.4	8.2 ± 3.8	1.5 ± 1.3
Kruskall Wallis test	3.63	0.46	3.34	0.41	0.66	2.06	1.01	2.26
*p*-value	0.163	0.796	0.188	0.815	0.718	0.357	0.603	0.323
According to children’s group								
ASD children	54.4 ± 15.1	0.7 ± 0.7	54.4 ± 15.1	1.1 ± 0.9	0.5 ± 0.7	0.4 ± 0.4	20.1 ± 11.3	1.2 ± 1.0
TD children	47.7 ± 10.7	0.9 ± 0.8	47.7 ± 10.7	4.8 ± 4.1	2.9 ± 2.9	0.7 ± 0.9	15.4 ± 4.8	1.6 ± 1.1
Mann Whitney-U test	176	208	176	219	222	194	219.5	210
*p*-value	0.137	0.347	0.137	0.072	0.142	0.938	0.066	0.273
